# Randomized Control Trial of Culturally Adapted Cognitive Processing Therapy for PTSD Substance Misuse and HIV Sexual Risk Behavior for Native American Women

**DOI:** 10.1007/s10461-018-02382-8

**Published:** 2019-01-03

**Authors:** Cynthia R. Pearson, Debra Kaysen, David Huh, Michele Bedard-Gilligan

**Affiliations:** 1 Indigenous Wellness Research Institute, School of Social Work, University of Washington, Seattle, USA; 2 Department of Psychiatry and Behavioral Sciences, University of Washington, Seattle, USA

**Keywords:** Cognitive processing therapy, PTSD, HIV/AIDS, American Indian and Alaska native, Substance misuse

## Abstract

An overlooked sequela of HIV risk is trauma exposure, yet few HIV interventions address trauma exposure, mental health, and substance misuse. In a two-arm randomized controlled trial 73 Native American women were randomized to a culturally-adapted Cognitive Processing Therapy (CPT) or 6-weeks waitlist. Outcomes assessed: PTSD symptom severity, alcohol use frequency, substance abuse or dependence diagnosis, and high-risk sexual behavior defined as vaginal/anal intercourse (a) under the influence of alcohol and/or illicit substances, (b) with a partner who was concurrently sexually active with someone else, and/or (c) with more than one partner in the past 6 weeks. Among immediate intervention participants, compared to waitlist participants, there were large reductions in PTSD symptom severity, high-risk sexual behavior, and a medium-to-large reduction in the frequency of alcohol use. CPT appears to improve mental health and risk behaviors, suggesting that addressing PTSD may be one way of improving HIV-risk related outcomes.

## Resumen

Una secuela pasada por alto acerca del riesgo de VIH es la exposición a trauma, sin embargo, pocas intervenciones del VIH abordan la exposición de trauma, la salud mental y el abuso de sustancias. En un ensayo controlado aleatorio de dos brazos, 73 mujeres nativas americanas fueron asignadas al azar a una Terapia de Procesamiento Cognitivo (TPC) adaptada culturalmente o a 6 semanas de espera. Resultados evaluados: Severidad de los síntomas de trastorno de estrés post-traumático (TEPT), frecuencia del consumo de alcohol, diagnóstico de abuso o dependencia de sustancias, y conducta sexual de alto riesgo definida como coito anal/vaginal a) bajo la influencia del alcohol y/o sustancias ilícitas, b) con una persona que tuvo relaciones sexuales concurrente con otras personas y/o c) con más de una persona en las últimas 6 semanas. Entre los participantes en la condición inmediata versus participantes en la lista de espera, hubo una gran reducción en la gravedad de los síntomas del TEPT, y reducción de conducta sexual de alto riesgo. Además, y una mediana a grande reducción en la frecuencia del consumo de alcohol. La TPC parece mejorar la salud mental y reducer las conductas de riesgo, lo que sugiere que tratar el TEPT puede ser una forma de mejorar las conductas relacionadas con el riesgo de VIH.

## Introduction

An important but overlooked sequela of trauma exposure is risk for HIV [1]. Trauma exposure also increases risk for post-traumatic stress disorder (PTSD) and substance misuse. These negative outcomes are interrelated as PTSD frequently co-occurs with alcohol and drug dependence (> 50% and > 30% respectively) [2], and in turn the co-occurrence reduces self-care practices and elevates HIV sexual-risk behavior. PTSD concurrent with HIV leads to more rapid HIV disease progression, poorer survival, greater cost in services, and lower adherence to medical treatment [3–5]. Although there are HIV interventions that address trauma exposure, mental health, and substance misuse among men who have sex with men [6–9], there are no interventions that address co-occurring PTSD, substance misuse, and HIV risk among American Indian and Alaska Natives (AIAN). HIV interventions that have failed to address PTSD among individuals with comorbid PTSD and HIV so had poorer condom use [10] and physical health [3] outcomes, and these outcomes were worse among those with PTSD [11, 12]. Thus, PTSD interventions may reduce ongoing substance use and HIV sexual risk behaviors [13, 14].

Exposure to trauma over the past several centuries has led to devastating consequences for AIAN health and wellness [15]. AIAN experience violence at a rate more than twice the national level (101 vs. 41/1000 persons) [16] and AIAN women are 2.5 times more likely to be raped or sexually assaulted than women from other racial backgrounds [17]. Given high rates of trauma exposure, it is not surprising that AIAN women have higher lifetime rates of PTSD than non-Native groups [18].

Trauma exposure and its impact on substance misuse and PTSD have received special attention from many AIAN communities [19]. Historical trauma—genocide, loss of land, cultural disruption, and child abuse and neglect—was common throughout colonization, including during the “boarding school era”, and had profound intergenerational health impacts on communities and individuals [20]. Although the vast majority of AIAN exposed to traumatic events demonstrate remarkable resilience [21], high rates of PTSD persist [22, 23]. Interventions specifically designed for AIANs exposed to trauma may hold promise for reducing substance misuse and HIV sexual risk behaviors [24]. However, few trauma-focused interventions for AIAN exist. Two recent reviews found no clinical trials addressing PTSD among AIAN [25, 26]. The National Registry of Evidence-based Programs and Practices (NREPP) in 2018, shows only one EBI addressing PTSD or trauma symptoms in an AIAN population (8%) while none were tested specifically within an AIAN population [27].

Culturally-tailored interventions are critical for this population, as tailored interventions may reduce high attrition of racial and ethnic minorities from programs developed for non-minority communities [28, 29]. Cognitive processing therapy (CPT) is a cognitive behavioral trauma-focused treatment that uses cognitive restructuring to resolve PTSD symptoms. CPT has been found to be effective in randomized clinical trials and efficacy studies across the US and internationally in low- and middle-income countries [30–35]. CPT is recommended as a front line evidence-based intervention for PTSD [36, 37].

This study’s primary objective was to assess the feasibility and effectiveness of CPT for AIAN woman with PTSD symptoms, high-risk sexual behavior, and substance use. Our study hypotheses were: (1) as compared to waitlist, improvements in PTSD, substance use, and HIV sexual-risk behavior will be greater at post intervention for those receiving immediate CPT; and (2) at 3 months post intervention, improvements in PTSD, substance use, and HIV sexual-risk behavior compared to pre-intervention will be greater for those receiving CPT compared to waitlist.

## Methods

### Setting and Recruitment

The study was conducted at two rural Pacific Northwest behavioral health clinics: a tribally-operated clinic located on the reservation and a privately-operated nonprofit clinic located in a town adjacent to the reservation. Our community assessment shown that 39.5% of women between the ages of 15 and 35 had high rates of mental health diagnoses (e.g., PTSD) [21, 38]. Recruitment materials were posted at tribal service centers, nearby colleges and churches, and distributed at local fairs and powwows. Potential participants were screened over the phone for eligibility by a clinical psychologist or social worker. Eligibility criteria were: (a) being an AIAN female ≥ 18 years of age; (b) at least 2 days of heavy drinking in the past year or any use of illicit substances in the last 3 months; (c) willingness to abstain from substance use during therapy; (d) a minimum of subthreshold PTSD symptoms [39] defined as meeting Criterion A and at least 2 of the B–E DSM-IV symptom criteria; [40, 41] and (e) self-reported sexual activity in the past 12-months. Exclusion criteria were (a) psychiatric medication changes or dose changes in the past 2 months; (b) presence of a psychotic disorder; (c) past 30-day suicide attempt, suicidal ideation with intent or plan, or self-harm, (d) past 3-month opioid use to ensure that participants are stable and are unlikely to need detoxification [42]. At the time of this study, it was recommended that individuals be free of opioids for at least 2 months prior to starting the trauma treatment [42]. As the study team did not include a licensed medical provider with expertise in opioid use disorders we chose a conservative range of 3 months. The study protocol was approved by the University of Washington Institutional Review Board (#43091, NCT01849029) and a tribal review board. Participants meeting eligibility criteria provided written consent. A data safety monitoring board comprised of a psychologist, psychiatrist, and a statistician monitored participants’ safety outcomes throughout the study. No serious adverse events were identified.

### Procedures

All participants completed 45-min audio computer-administered assessments at baseline, immediate post-, and 3-month post-intervention. Following baseline assessment, participants were randomly assigned to either receive immediate CPT or to a 6-week waitlist. At the end of the waitlist period, participants completed a post-waitlist assessment. Random assignment to condition was computer-generated with block sizes ranging from 2 to 8 to reduce the detection of a pattern and prepared by an external statistician. Allocation concealment involved the use of sequentially numbered, opaque, sealed envelopes containing the group assignment, which the research manager opened at the moment of randomization. Due to the nature of the intervention, participants and the study team could not be blinded to condition. Participants were compensated for each interview: baseline ($30), waitlist ($35, 6-week post baseline), post-intervention ($40 approximately 10 weeks after first session), and three-month post-intervention ($50). Travel was reimbursed up to $20.00 for each intervention session (up to $260 total).

### Intervention

Based on community feedback we used CPT without the trauma narrative [43]. In CPT the therapist and client work collaboratively to identify maladaptive and inaccurate beliefs related to the trauma, specifically emphasizing distorted beliefs about the cause of the event (e.g., self-blame), and overgeneralized beliefs about self, others, and the world. Over the course of treatment, the client is taught to challenge their own beliefs and patterns of thinking to be more realistic and adaptive.

### Adaption of CPT

We followed the *Map of Adaptation Process*, which is a systematic approach for adapting evidence-based behavioral interventions (EBIs) [44]. The adaptation process is described in full in [45]. Here we provide a brief summary. Over 2 years, in full collaboration with a tribal advisory board, we adapted CPT for use in this AIAN community. We removed barriers for community providers in using the CPT manual by: removing scientific jargon; improving readability; and culturally adapting concepts, definitions, and handout materials. Specifically, the adapted CPT manual, as compared to the original CPT manual, had fewer total pages (110 vs. 213) and sentences per paragraph (1.6 vs. 2.4). We improved readability by reducing the percentage of passive sentences (7% vs. 13%) and Flesch-Kincaid grade level (7.7 vs. 12.6), and increased the Flesch reading ease (66.7% vs. 30.5%).

The manual’s content closely resembles the original CPT manual, however based on community input we added a pre-session to promote engagement and therapy rationale. We also added content on relationships, safer sex behaviors, and substance use. Thus, the CPT protocol used here included 13 sessions: (1) introduction—trauma and its effects on behavior; (2) identifying the worst trauma and review of trauma symptoms; (3) meaning of the event; (4) connections among events, thoughts, and feelings; (5) thinking questions; (6) addressing “Unhelpful” thinking patterns; (7) “Balancing” thoughts and feelings; (8) safety; (9) trust; (10) power and control; (11) respect; (12) sexual behavior and substance use; and (13) caring, intimacy, and a closing ceremony (the adapted manual is available from the corresponding author). In general, we replaced clinical examples and skill building exercises with ones relevant to the AIAN community (i.e., removing the combat examples and providing discussion of childhood abuse, gun violence, and substance use-related accidents in an AIAN setting). The last 3 modules included additional content to address relationships, substance use, and safer sex [46]. We incorporated indigenous beliefs reflective of the community values pertaining to spirituality, death, family, tribal specific historical trauma, and role of elders and cultural activities as support networks. The CPT skill building handouts, tips for counselor-client relationship, and service delivery were altered to provide local indigenous images and protocols such as smudging, understanding of seasonal ceremonies and memorials.

### Counselor Training and Monitoring

Counselors successfully completed 1 week of training before delivering CPT and attended weekly supervision meetings with a clinical psychologist with CPT expertise throughout the study duration. Supervision calls included monitoring of weekly symptom measures, review of audio recordings of intervention sessions, and group discussion on case conceptualization, delivery of strategies, and clinical challenges. The calls were intended to ensure fidelity and adherence to CPT and to prevent counselor drift in delivering the intervention.

#### Measures

Most items and all scales were selected from published and validated measures, and all items were pretested for cultural appropriateness in this community.

#### PTSD Symptoms

The 17-item PTSD Symptom Scale Self-Report Version [47] was used to assess the presence and severity of past month PTSD symptoms based on DSM-IV criteria. Participants were asked to rate the frequency and intensity of each of the DSM-IV PTSD symptoms (e.g., “Having upsetting thoughts or images about the traumatic event that came into your head when you didn’t want them to?”). The response options included 0 = *Not at all*, 1 = *Once per week or less/A little*, 2 = *2 to 4 times per week/Somewhat*, and 3 = *5 or more times per week/Very much*. Participant responses were summed to create a total severity score of PTSD symptoms ranging from 0 to 51, with higher scores reflecting greater severity of PTSD symptoms (published α = 0.85, study α = 0.91) [47].

#### Alcohol-Related Problems

The 15-item *Alcohol Short Inventory of Problems* [48] was used to assess the physical, social, intrapersonal, impulsive, and interpersonal consequences of past year alcohol consumption for the baseline assessment or since the last assessment for all follow-ups. Statements included, “I have been unhappy because of my drinking/drug use,” “Because of my drinking/drug use, I have not eaten properly.” The fifteen responses (0 = *No* or 1 = *Yes*) were summed to create an index of alcohol-related problems ranging from 0 to 15, with higher scores reflecting greater severity of problems (published α = 0.98, study α = 0.95) [48].

#### Alcohol Use

A *Drug Use Frequency* measure [49] assessed the frequency of alcohol use. The frequencies of consuming beer, malt liquor or cider, alcoholic energy drinks, liquor, and/or wine consumption were assessed in five separate items (e.g., “How often did you drink beer during the past 6 weeks?”). Response options for each item included 1 = *never*, 2 = *several times*, 3 = *about once a month*, 4 = *several times a month*, 5 = *1*–*2* *days a week*, 6 = *3*–*4* *days a week*, 7 = *5*–*6* *days a week*, and 8 = *every day*. The maximum frequency of alcohol consumption across the 5 items was used as an index of the frequency of alcohol use, with higher scores reflecting greater alcohol use.

#### Substance Use Disorder

The Mini International Neuropsychiatric Interview for DSM-IV (MINI) [50] was used to identify the presence or absence of non-alcohol substance abuse or dependence based on DSM-IV criteria across eight categories of substance use: (a) cocaine, (b) hallucinogens, (c) narcotics, (d) stimulants, (e) inhalants, (f) tranquilizers, (g) marijuana, and/or (h) other illicit drugs. If a participant met DSM-IV criteria for substance abuse or dependence in one or more substance categories, they were coded as having a substance use disorder.

#### High-risk Sexual Behavior

Four items assessed engagement in high-risk sexual behaviors [51] including unprotected vaginal/anal intercourse while (a) under the influence of alcohol, (b) under the influence of illicit substances, (c) with a partner who was sexually active with someone else, and/or (d) with more than one partner in the past 6 weeks. Items included “In the last 6 weeks how often did you drink alcohol before having vaginal or anal intercourse with any of your partners?” and “In the last 6 weeks were you with a partner who was having sex with someone else besides you?” For consistency, items assessing frequency were re-coded as dichotomous variables (i.e., 0 = *No*, 1 = *Yes*) and summed to create an overall index of high-risk sexual behavior ranging from 0 to 4, with higher scores reflecting greater high-risk sexual behavior.

Unprotected sex in the past 6 weeks was calculated using two items assessing (1) the total number of vaginal/anal intercourse acts and (2) the number of condom-protected sex acts. First, the number of unprotected sex acts was calculated by subtracting the number of times condoms were used from total number of vaginal/anal intercourse acts. Second, the proportion of non-condom protected sex acts was calculated by dividing the number of unprotected sex acts by the total number of sex acts.

### Data Analysis

#### Main Outcome Analyses

To evaluate the efficacy of the CPT intervention versus the waitlist control, longitudinal regression analyses were conducted using generalized estimating equations (GEE) [52] with cluster robust standard errors [53]. All participants randomized at baseline were included in the primary outcome analyses (i.e., an intent-to-treat approach) using all available data, including participants who missed one or more assessments. The primary outcome was (a) PTSD symptoms, and the secondary outcomes were (b) alcohol-related problems, (c) alcohol use frequency, (d) substance use disorder, (e) total high-risk sexual behaviors, and (f) percentage of non-condom protected vaginal/anal intercourse acts. All study outcomes were chosen a priori. Gaussian GEE models were used for relatively normally distributed variables and logistic GEE models [54] for outcomes bounded between 0 and 1. All data analyses were conducted in R version 3.5.1 with version 4.13–19 of the *gee* package [55, 56].

For the main outcome analyses, each outcome was regressed on Treatment (CPT vs. Waitlist control), Time (Post-intervention vs. baseline), and the Treatment by Time interaction. For the non-condom protected vaginal/anal intercourse outcome, having a primary partner versus no primary partner was included as an a priori covariate. The statistical test of the intervention effect was the CPT × time interaction.

#### Quasi-experimental Intervention Analyses

We conducted secondary analyses to evaluate the effectiveness of the CPT intervention among participants initially assigned to the waitlist control condition. A piecewise linear approach was used to model the difference in outcome trajectories during a) the waitlist phase (0 to 6 weeks), b) the intervention phase (6 to 12 weeks), and c) the follow-up phase (12 to 24 weeks). Each outcome was regressed on time, which was divided into three linear parameters corresponding with (a) the rate of change during the waitlist period, (b) the change in trajectory after introduction of the intervention (i.e., the quasi-experimental intervention effect), and (c) the change in trajectory after completion of the intervention.

#### Dose Response

Dose response analyses were conducted to evaluate the association between (a) number of CPT sessions attended and (b) post-baseline change in primary outcomes among all 73 participants. In the dose–response analyses, each outcome was regressed on the dose variable, time, and the dose by time interaction. Time was coded as a dichotomous contrast of pre-intervention versus post-intervention/follow-up. The magnitude and statistical significance of the dose by time interaction evaluated whether the number of sessions attended moderated change in the intervention outcome from pre- to post-intervention/follow-up.

## Results

### Flow of Participants

**Fig. 1:**
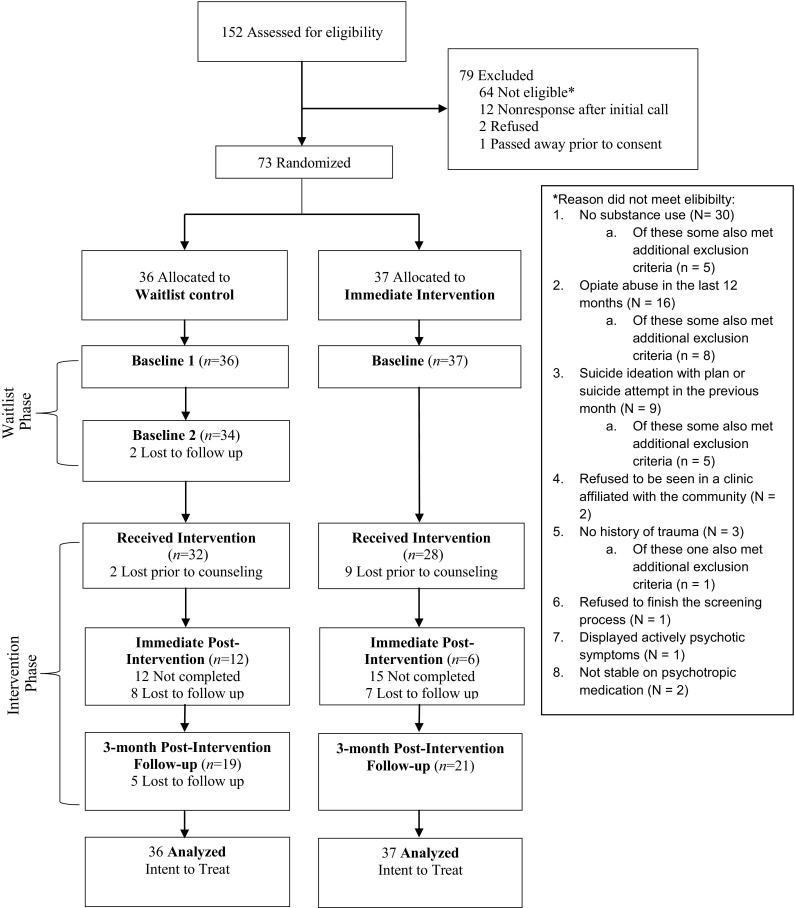
Participant flow chart

As seen in Fig. 1, of the 73 participants randomized to the waitlist control (*n *= 36) or immediate intervention conditions (*n *= 37), 60 received at least one session (32 waitlist arm, 28 immediate arm).

### Participants

Sociodemographic and other descriptive information for the final analytic sample of 73 female participants by study condition are provided in Table 1.

**Table 1:** Baseline characteristics of participants overall and by study condition

	All participants (*N* = 73)		Waitlist control (*n* = 36)		Immediate arm (*n* = 37)	
	*n*	*%*	*n*	*%*	*n*	*%*
Age in years						
18 to 29	16	21.9	8	22.2	8	21.6
30 to 39	21	28.8	10	27.8	11	29.7
40 to 49	23	31.5	13	36.1	10	27.0
50 to 60	13	17.8	5	13.9	8	21.6
Education						
Less than HS	27	37.0	12	33.3	15	40.5
HS graduate	25	34.2	12	33.3	13	35.1
AA degree and higher	21	28.8	12	33.3	9	24.3
Employment						
Unemployed	59	80.8	31	86.1	28	75.7
Part-time employed	4	5.5	1	2.8	3	8.1
Full-time employed	10	13.7	4	11.1	6	16.2
Primary partner						
No	33	45.2	16	44.4	17	45.9
Yes	40	54.8	20	55.6	20	54.1
PTSD diagnosis						
No	25	34.2	12	33.3	13	35.1
Yes	48	65.8	24	66.7	24	64.9
Substance use disorder						
No	22	30.1	10	27.8	12	32.4
Dependence Dx	3	4.1	2	5.6	1	2.7
Abuse Dx	48	65.8	24	66.7	24	64.9

*HS* high school, *AA* associates, *PTSD* post-traumatic stress disorder, *Dx* diagnosis

### Missing Data

With respect to the primary study outcomes, 21% of participants (*n *= 15) had complete data from all assessments (immediate intervention: 3 assessments; waitlist control: 4 assessments), 42% (*n *= 31) were missing one assessment, and 37% were missing multiple assessments (*n *= 27). To assess for differences between participants who completed all assessments and those who were missing a single or multiple assessments, Pearson χ^2^ tests and one-way analyses of variance were conducted, respectively, on categorical and continuous socio-demographic characteristics and baseline levels of the outcomes. There were no statistically significantly differences between the degree of missing data and any demographic characteristic or baseline outcome.

### Intervention Participation

Most participants (82%, *n *= 60) attended at least one counseling session. Those that attended counseling completed an average of 6.1 (*SD *= 4.4) sessions with attendance ranging from 1 to 13 sessions; 30% of the participants that attended counseling completed treatment, defined as attending at least nine sessions.

### Primary Outcome Analyses

**Fig. 2:**
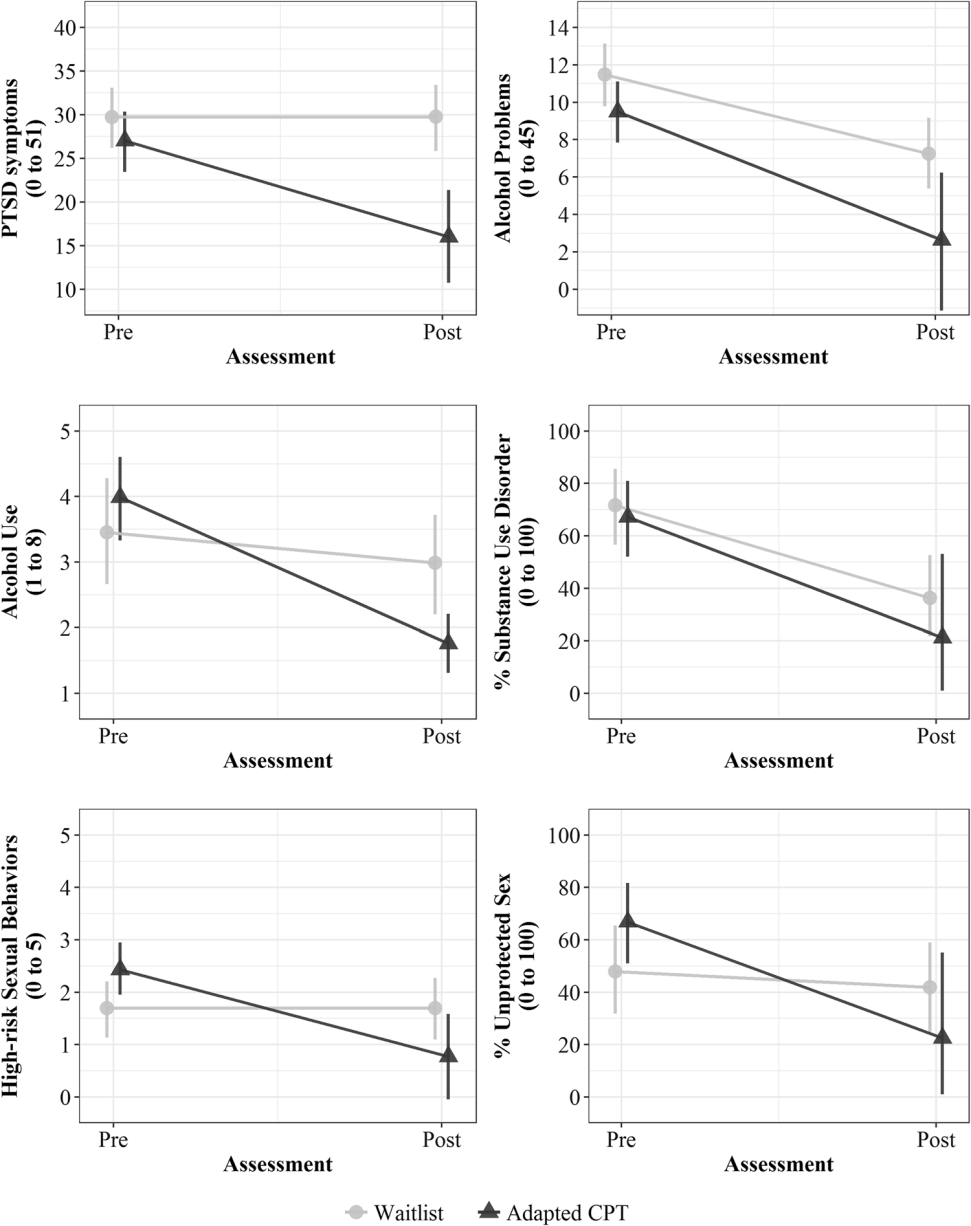
Predicted means of study outcomes at pre- and post-intervention/follow-up by immediate intervention versus waitlist control condition (*N* = 73)

Table 2 provides descriptive statistics for each of the primary outcomes along with the magnitude and statistical significance of the intervention effects (Cohen’s *d*). There were 3 significant intervention effects among the 6 outcomes. Among immediate intervention participants compared to waitlist control participants there was a large reduction in PTSD symptom severity (*d *= 1.03, *p *< 0.001), frequency of alcohol use (*d *= 0.77, *p *= 0.002) and sexual risk behaviors (*d *= 1.02, *p *= 0.004). There were no significant differences between immediate intervention and waitlist control participants with respect to post-baseline change in alcohol problems, illicit substance use, and rates of non-condom protected sex. Figure 2 depicts the predicted mean outcomes by time and study condition based on the primary intervention analyses.

**Table 2:** Study outcomes by condition and primary outcome analysis estimates

	Scale range	Waitlist control				Immediate intervention			Intervention effect	
		BL (*n *= 36)	Pre (*n *= 34)	Post (*n *= 12)	FU (*n *= 19)	Pre (*n *= 37)	Post (*n *= 6)	FU (*n *= 21)	*d*	*P*
		Mean (SD)	Mean (SD)	Mean (SD)	Mean (SD)	Mean (SD)	Mean (SD)	Mean (SD)		
Primary outcome										
PTSD symptoms	[0, 51]	29.7 (10.7)	30.1 (11.7)	22.5 (14.4)	20.0 (13.0)	27.1 (10.8)	14.7 (8.4)	19.0 (9.7)	**1.03**	**< 0.001**
Secondary outcome										
Alcohol problems	[0, 45]	11.5 (5.1)	7.3 (5.6)	5.0 (6.6)	5.1 (5.9)	9.5 (5.1)	2.8 (4.3)	5.6 (6.0)	0.50	0.267
Alcohol use	[1,8]	3.4 (2.5)	3.0 (2.3)	3.1 (2.6)	3.5 (2.4)	4.0 (2.0)	1.2 (0.4)	2.1 (1.8)	**0.77**	**0.002**
% Substance use disorder	[1, 100]	72.2 (45.4)	35.3 (48.5)	33.3 (49.2)	30.0 (47.0)	67.6 (47.5)	16.7 (40.8)	18.2 (39.5)	0.44	0.485
High-risk sexual behavior	[0, 4]	1.7 (1.7)	1.7 (1.8)	2.1 (2.0)	1.6 (1.8)	2.4 (1.5)	0.5 (1.2)	2.0 (1.7)	**1.02**	**0.004**
% Unprotected sex	[0, 100]	48.8 (49.8)	42.2 (49.4)	57.5 (50.8)	41.1 (46.7)	64.4 (46.1)	16.7 (40.8)	52.9 (49.7)	1.07	0.086

Statistically significant intervention effects highlighted in bold. A positive intervention effect corresponds with better outcomes among immediate intervention participants

*BL* waitlist baseline, *Pre* pre-intervention baseline, *Post* immediate post-intervention, *FU* follow up, *d* Cohen’s *d*, *SD* standard deviation

### Secondary Quasi-experimental Outcome Analyses

The magnitude and statistical significance of the quasi-experimental intervention effects are provided in Table 3 and summarize the change in outcomes during each of the study phases (waitlist, intervention, follow up) and the difference in change from (a) the waitlist phase to intervention phase and (b) the intervention phase to follow-up phase, among those initially randomized to waitlist control. Across the 6 outcomes, there was one quasi-experimental intervention effect that was statistically significant. Specifically, PTSD symptoms were mostly unchanged during the waitlist phase (*d *= − 0.01, *p *= 0.886), but improved significantly during the intervention phase (*d *= 0.68, *p *= 0.015), a medium to large quasi-experimental intervention effect (*d *= 0.69, *p *= 0.002).

**Table 3:** Summary of quasi-experimental intervention effects (*n *= 36)

	Within-phase change						Between-phase change			
	Waitlist phase		Intervention phase		Follow up phase		Waitlist to intervention		Intervention to follow up	
	*d*	*p*	*d*	*p*	*d*	*p*	*d*	*p*	*d*	*p*
Primary outcome										
PTSD symptoms	− 0.01	0.886	**0.68**	**0.015**	0.19	0.313	**0.69**	**0.042**	− 0.49	0.365
Secondary outcome										
Alcohol problems	**0.83**	**< 0.001**	0.41	0.178	− 0.03	0.907	− 0.41	0.394	− 0.44	0.396
Alcohol use	0.19	0.310	0.06	0.901	− 0.19	0.425	− 0.12	0.753	− 0.26	0.613
Substance use disorder (%)	**0.80**	**< 0.001**	0.03	0.923	0.04	0.904	− 0.78	0.073	0.01	0.984
High-risk sexual behavior	− 0.01	0.934	− 0.09	0.797	0.16	0.424	− 0.09	0.757	0.25	0.487
Unprotected sex (%)	0.14	0.591	− 0.10	0.671	0.30	0.332	− 0.24	0.479	0.40	0.405

A positive effect size corresponds with improvement in outcomes. Statistically significant estimates highlighted in bold

### Dose Responses Analyses

Across the 6 outcomes, there were two statistically significant dose–response effects. Each additional CPT session attended was associated with a 1.3-point greater reduction in PTSD symptoms (*Est *= − 1.32, 95% CI [− 1.93, − 0.70]) and a 0.4-point greater reduction in alcohol problems (*Est *= − 0.40, 95% CI [− 0.77, − 0.03]).

## Discussion

Our findings support the efficacy of CPT, a manualized trauma-focused therapy, adapted for use with AIAN women for treatment of PTSD, substance use, and high risk sexual behavior. Given the high level of trauma exposure and related symptomatology among AIAN communities [16, 57] and the paucity of clinical trials research on AIAN women [58] findings are promising. CPT had large effects on improving PTSD and high risk sexual behavior and moderate to large effects on alcohol use. Although exposure-based PTSD treatments have been evaluated for those with comorbid alcohol and substance use disorders [59, 60], this is the first clinical trial of a cognitively based trauma-focused therapy for reducing PTSD, substance use and HIV risk [61].

Consistent with hypotheses, CPT had large and statistically significant effects in improving PTSD symptoms, both from pre- to post-treatment and between randomization conditions. Among participants in the immediate intervention group, average PTSD symptom scores dropped by nearly half, whereas waitlist control participants maintained the same PTSD symptoms, on average, during their waitlist period. In secondary analyses, we found that after waitlisted participants received the intervention, they had comparable reductions in PTSD symptoms to their immediate intervention counterparts. Despite these positive findings, it is important to note that post-treatment PTSD symptoms remained at a moderate level, higher than what is typically seen following CPT and other evidence-based PTSD interventions. In CPT studies, typically only 20% of participants continue to meet diagnostic criteria for PTSD following treatment [62]. One explanation for our lower than established reduction in PTSD is that the AIAN community experiences ongoing racism, ranging from micro-aggression to overt discrimination and assault which may have negatively impacted recovery [63]. A prior study found, however, comparable levels of improvement with CPT for African Americans, despite higher drop-out and lower rates of therapy initiation [64].

Our findings on the effectiveness of CPT on substance use and high risk sexual behavior are promising. The HIV prevention literature has emphasized the importance of reducing substance use and addressing traumatic stress to reduce high risk sexual behaviors, especially for women [10, 65, 66]. Reductions in trauma symptoms have been linked to decreases in sexual risk behavior [67, 68] although interventions tested to date have not directly addressed PTSD. In contrast, PTSD interventions for individuals with comorbid substance use disorders have generally found significant improvements in PTSD, although findings are inconsistent regarding their effects on substance use outcomes [59, 60]. We found that CPT was associated with medium-to-large reductions in alcohol consumption and in sexual risk behaviors. We did not find differences in alcohol-related consequences, frequency of illicit substance use, or rates of non-condom protected sex. These findings suggest that one route to helping improve sexual risk and substance use outcomes, specifically alcohol use, for AIAN may be through addressing PTSD and trauma.

Our findings also highlight the importance of treatment dose, suggesting that the more individuals participate in a structured therapy, the better their treatment gains. Specifically, attending more sessions of CPT was associated with lower PTSD symptoms and alcohol problems. Treatment drop-out was high, although it was comparable with other PTSD/SUD trials which tend to have around 50–60% rates of dropout [69]. This highlights the importance of finding shorter interventions for treatment of PTSD/SUD. Especially in communities with high rates of poverty and stress and with lower levels of education, the typical length of treatment in many PTSD/SUD interventions may be too long and individuals may receive inadequate “dosing” of therapy [70].

It is also important to note that one major point of drop-out for our study was between the initial assessment and the first session of psychotherapy, with almost a quarter (21.7%) of clients never receiving any CPT. The modal number of therapy sessions in clinical settings is one [71]. This may be a wasted opportunity, as individuals present hoping for care and resources, which may not be provided during this first session. As a field it may be worthwhile re-evaluating how we structure client experiences during intake and how to improve the handoff to active clinical care and treatment.

This study has several limitations. With over 567 federal and 200 state-recognized tribes as well as multiple unique urban AIAN communities, our Pacific Northwest regional focus may not fully capture AIAN retention and response to CPT. In addition, we did not utilize independent, blinded interviewer administered assessments, which are the recommended method of assessing treatment response [72]. However, the advantages of ACASI assessments include: privacy in responding to sensitive questions, elimination of data entry time and errors, and consistent questionnaire delivery. Finally, interpretation of findings is limited by small sample sizes and high attrition from both active treatment and post-treatment assessments, although effect sizes were moderate to large and symptom changes occurred in the hypothesized direction for all outcomes suggesting a clinically meaningful effect of treatment. The inability to retain the sample is in and of itself an important finding of the study as it suggests the need for brevity, simplicity, and reduction of treatment barriers in this population.

Overall, results of this study suggest that providing a short-term, structured, culturally adapted intervention for PTSD can decrease PTSD, substance use, and HIV risk behaviors in a sample of AIAN women. Given the high rates of trauma exposure and associated negative outcomes in AIAN communities this study offers a potential efficacious option for addressing an important public health disparity. Future studies should expand on this work by exploring briefer therapy protocols to help improve retention and engagement. Addressing trauma is a community driven and crucial priority for the health and prosperity of AIAN communities and continues to be an area of great importance.
